# Comparative predictive performance of different eGFR equations for 1-year major adverse cardiovascular events after percutaneous coronary intervention in patients with acute coronary syndrome

**DOI:** 10.3389/fcvm.2026.1863081

**Published:** 2026-09-09

**Authors:** Yicheng Wang, Fangming Liu, Ruoxi Qi, Xiaohan Wang, Jiajie Mei, Zhenzhu Liu, Lijiao Zhang, Wenli Xie, Ming Yu, Xiaodong Zhang, Hongyan Wang, Zhaohong Geng

**Affiliations:** 1 Department of Cardiology and the Institute of Heart and Vascular Diseases, The Second Affiliated Hospital of Dalian Medical University, Dalian, Liaoning, China; 2 College of Basic Medical Science, Dalian Medical University, Dalian, Liaoning, China; 3 Department of Mental Health and Psychology, Dalian Medical University, Dalian, Liaoning, China; 4 College of Sports Medicines and Rehabilitation, Shandong First Medical University, Taian, Shandong, China

**Keywords:** eGFR, MACE, machine learning, PCI, prediction

## Abstract

**Background:**

Renal dysfunction is an important determinant of cardiovascular prognosis in patients with coronary artery disease. Estimated glomerular filtration rate (eGFR) is widely used to assess kidney function, but different equations may yield varying risk stratification results. This study aimed to compare the predictive performance of creatinine-based and cystatin C–based eGFR equations for 1-year major adverse cardiovascular events (MACE) in patients undergoing percutaneous coronary intervention (PCI).

**Methods:**

This retrospective study included 560 patients who underwent PCI for acute coronary syndrome. Baseline clinical, laboratory, and echocardiographic variables were collected. Renal function was assessed using both creatinine-based and cystatin C–based eGFR equations. Logistic regression with backward selection based on the Akaike Information Criterion (AIC) was performed to identify independent predictors of 1-year MACE. In addition, multiple machine learning models were developed to evaluate predictive performance, and Shapley Additive Explanations (SHAP) analysis was applied to assess feature importance.

**Results:**

During the 1-year follow-up, MACE occurred in 72 patients, comprising 71 repeat PCIs and one cardiac death. Equations relying on cystatin C detected a larger share of patients with impaired renal function compared with creatinine-based formulas. However, moderate-to-severe renal dysfunction defined by creatinine-based eGFR was significantly associated with an increased risk of 1-year MACE, although it was not retained as an independent predictor after AIC–based multivariable adjustment. Machine learning models demonstrated modest predictive performance overall, with logistic regression achieving the highest discriminative ability. SHAP analysis indicated that cardiovascular functional parameters and metabolic markers were the major contributors to risk prediction, while renal function indicators provided complementary prognostic information.

**Conclusions:**

Creatinine-based eGFR showed a stronger observed association with 1-year MACE after PCI than cystatin C–based eGFR in this single-center cohort; these findings do not establish clinical superiority of either equation and require external validation. Renal function assessment remains an important component of cardiovascular risk stratification, and traditional regression models performed comparably to machine learning approaches in moderately sized clinical datasets.

## 1 Introduction

Percutaneous coronary intervention (PCI) has become a cornerstone therapy for patients with coronary artery disease and plays a crucial role in improving myocardial perfusion and clinical outcomes (1, 2). Despite continuous advances in interventional techniques, stent technology, and pharmacological therapies, a considerable proportion of patients still experience major adverse cardiovascular events (MACE) during follow-up (3). These events, including repeat PCIs and cardiac death, remain major determinants of long-term prognosis after PCI (4). Therefore, early identification of patients at high risk for adverse cardiovascular outcomes is essential for optimizing post-procedural management and improving long-term survival.

Renal dysfunction is widely recognized as an important risk factor for cardiovascular disease and adverse clinical outcomes (4). Reduced kidney function is associated with multiple pathophysiological processes that contribute to cardiovascular complications, including endothelial dysfunction, chronic inflammation, oxidative stress, and accelerated atherosclerosis (5–7). Previous studies have consistently demonstrated that impaired renal function is associated with increased risks of mortality and cardiovascular events in patients with coronary artery disease (4). In the setting of PCI, patients with reduced glomerular filtration rate often exhibit more complex coronary lesions, greater comorbidity burden, and poorer clinical outcomes (8, 9). Consequently, accurate assessment of renal function plays a critical role in cardiovascular risk stratification in patients undergoing coronary revascularization.

Estimated glomerular filtration rate (eGFR) is the most commonly used clinical indicator for evaluating renal function (10). Traditionally, eGFR has been calculated using serum creatinine-based equations (11). However, creatinine levels can be influenced by several non-renal factors, such as muscle mass, age, diet, and sex, which may compromise the accuracy of renal function estimation in certain populations. In recent years, cystatin C has emerged as an alternative biomarker for estimating kidney function (12, 13). Because cystatin C is produced by all nucleated cells and is less affected by muscle mass and dietary intake, cystatin C–based eGFR equations have been proposed to provide a more sensitive assessment of renal impairment. Several studies have suggested that cystatin C may identify early kidney dysfunction that is not detected by creatinine-based measures (12, 14, 15). Nevertheless, the comparative prognostic value of creatinine-based and cystatin C–based eGFR equations for predicting cardiovascular outcomes has not been fully established. In particular, evidence comparing these two approaches in patients undergoing PCI is still limited.

Given the growing interest in precision risk prediction, machine learning methods have also been increasingly applied in cardiovascular research to improve outcome prediction by capturing complex relationships among clinical variables (16, 17). However, whether advanced machine learning approaches provide incremental predictive value beyond conventional statistical models in this clinical context remains uncertain.

Therefore, the present study aimed to compare the performance of creatinine-based and cystatin C–based eGFR equations in predicting 1-year MACE in patients undergoing PCI. Furthermore, both traditional logistic regression and machine learning models were employed to assess the relative contribution of renal function indicators and other clinical variables to risk prediction. By integrating conventional statistical analysis with modern predictive modeling, this study sought to provide a comprehensive assessment of the prognostic value of different eGFR equations in patients undergoing PCI.

## 2 Methods

### 2.1 Study population

This study was designed as a retrospective cohort study. The study population consisted of consecutive adult patients with coronary artery disease who were admitted to the Second Affiliated Hospital of Dalian Medical University between March 2023 and September 2023 and underwent PCI. All patients underwent PCI for acute coronary syndrome (ACS), including unstable angina, ST-elevation myocardial infarction, or non-ST-elevation myocardial infarction. After applying the predefined exclusion criteria, 560 patients were included in the final analysis. Coronary artery disease was confirmed by coronary angiography demonstrating at least 50% stenosis in one or more major coronary arteries, including the left main coronary artery, left anterior descending artery, left circumflex artery, or right coronary artery. Significant coronary stenosis was defined as ≥70% diameter stenosis in non-left-main vessels or ≥50% stenosis in the left main coronary artery, and the decision to perform PCI was made according to current clinical guidelines and the judgment of experienced interventional cardiologists.

Clinical data were extracted from the electronic medical record (EMR) system of the Second Affiliated Hospital of Dalian Medical University. The study protocol was approved by the Ethics Committee of the Second Affiliated Hospital of Dalian Medical University (Approval No.: KY2025-142-01) and was conducted in accordance with the principles of the Declaration of Helsinki. Given the retrospective nature of the study, the requirement for informed consent was waived.

Patients were excluded if they met any of the following conditions:

1. Severe valvular heart disease, malignant arrhythmia, advanced heart failure, congenital heart disease, hypertrophic obstructive cardiomyopathy, or cor pulmonale;
2. Previous history of coronary artery bypass graft surgery;
3. Presence of hematologic diseases, malignancy, or hyperthyroidism;
4. Current treatment with systemic corticosteroids;
5. Receipt of dialysis therapy;
6. Presence of acute kidney injury (AKI) at the time of admission;
7. Missing values in any variable required for the present analysis. Patients with missing data for any study variable included in the analysis were excluded during eligibility screening. As a result, the final analytical cohort consisted of 560 patients with complete data for all study variables (0% missingness), and no imputation was required.

### 2.2 Data collection and definition

Baseline clinical data, including demographic characteristics, medical history, laboratory findings, and echocardiographic parameters, were retrospectively collected from the EMR system. Demographic and clinical variables included age, sex, body mass index (BMI), hypertension, diabetes mellitus, smoking status, alcohol consumption, history of stroke, and the number of diseased coronary vessels. All patients received a standardized dual antiplatelet therapy (DAPT) regimen following PCI.

Blood samples were collected from the antecubital vein at the last measurement obtained prior to PCI and analyzed in the central laboratory of the hospital using an automated biochemical analyzer. Laboratory parameters included serum creatinine, cystatin C, blood urea nitrogen, creatine kinase-MB (CK-MB), glycated hemoglobin, triglycerides, hemoglobin, alanine aminotransferase, and aspartate aminotransferase. Serum creatinine was measured using an enzymatic (sarcosine oxidase) method [Creatinine Kit (CR); Meikang Biotechnology Co., Ltd., Ningbo, China], and serum cystatin C was measured using a latex-enhanced immunoturbidimetric assay [Cystatin C Kit (CYS-C); same manufacturer], both performed on the hospital's routine clinical chemistry analyzer and calibrated according to the manufacturer's instructions using the manufacturer-supplied calibrators, in accordance with the laboratory's standard operating procedures. Echocardiographic measurements, including left ventricular end-diastolic diameter and left ventricular ejection fraction, were obtained using standard transthoracic echocardiography.

Renal function was assessed using eGFR. Two different equations were applied to calculate eGFR: a creatinine-based equation and a cystatin C–based equation.

The creatinine-based equation was calculated as follows (18):

$$\mathrm{eGFR}=186\times Cr^{-1.154}\times \mathrm{Ag}e^{-0.203}\times 1.233\times (0.742\;\mathrm{if}\;\mathrm{female}).$$

For the cystatin C–based equation (19):

IfCysC≤0.8mg/L

$$\mathrm{eGFR}=133\times (\mathrm{CysC}/0.8{)}^{-0.499}\times 0.996^{\mathrm{Age}}\times (0.932\;\mathrm{if}\;\mathrm{female})$$

IfCysC>0.8mg/L

$$\mathrm{eGFR}=133\times (\mathrm{CysC}/0.8{)}^{-1.328}\times 0.996^{\mathrm{Age}}\times (0.932\;\mathrm{if}\;\mathrm{female}).$$

According to the Kidney Disease: Improving Global Outcomes (KDIGO) guidelines, renal function was categorized into three groups: normal (eGFR ≥ 90 mL/min/1.73 m^2^), mildly decreased (60 ≤ eGFR < 90 mL/min/1.73 m^2^), and moderate-to-severely decreased (eGFR < 60 mL/min/1.73 m^2^). This simplified three-category scheme, rather than the full KDIGO G1–G5 classification, was adopted because only 72 MACE events occurred during follow-up; subdividing the eGFR < 60 mL/min/1.73 m^2^ group further into the KDIGO G3b–G5 categories would have produced strata with too few events for stable estimation. The primary outcome of this study was the occurrence of MACE within 1 year after PCI. MACE was defined as a composite endpoint comprising cardiac death and repeat coronary revascularization. Cardiac death was defined as death occurring during the follow-up period that was attributed to a cardiovascular cause. Repeat PCI was defined as any unplanned repeat percutaneous coronary intervention performed after the index procedure during the 1-year follow-up period. Other adverse events identified during follow-up, including arrhythmia, angina pectoris, and heart failure, were recorded separately and were not counted as components of the composite MACE endpoint in this study. One-year outcome ascertainment was performed through a combination of telephone contact with patients or their family members and review of electronic medical records for subsequent clinic visits or hospital readmissions.

### 2.3 Statistical analysis

Descriptive statistics, between-group comparisons, and logistic regression analyses were performed with R (version 4.4.1), while all remaining analyses were performed with Python (version 3.13). Conventional statistical analyses were conducted in R, whereas machine learning models were implemented in Python. All statistical tests were two-sided, and a *p*-value < 0.05 was considered statistically significant.

Continuous variables were summarized as median with interquartile range (IQR). Group comparisons were conducted using the Wilcoxon rank sum test (Mann–Whitney *U* test) or the Kruskal–Wallis test where appropriate. Categorical variables were presented as frequencies and percentages and compared using Pearson's chi-square test or Fisher's exact test when the expected cell count was fewer than five. Univariable logistic regression analyses were first performed to evaluate the association between each candidate variable and the risk of 1-year MACE. All these variables were subsequently entered into a multivariable logistic regression model to identify independent predictors. A backward stepwise selection procedure based on the Akaike Information Criterion (AIC) was applied using the stepAIC function from the MASS package in R to determine the optimal combination of predictors. Odds ratios (ORs) and 95% confidence intervals (CIs) were estimated using the Wald method.

Given the limited number of events relative to the number of candidate predictors, penalized logistic regression (LASSO and elastic-net) was additionally performed in parallel with the backward stepwise model, as a complementary approach to evaluate the stability of the identified associations. The number of candidate predictors used in univariable analysis, the number of predictors retained in the final stepwise model, and the corresponding events-per-variable (EPV) ratio were calculated. Penalized logistic regression models [least absolute shrinkage and selection operator (LASSO) and elastic-net, with the elastic-net mixing parameter set at 0.5] were fitted using the same candidate predictor set, with the regularization parameter selected via 10-fold cross-validation to maximize the area under the receiver operating characteristic curve (AUC); predictors whose coefficients were shrunk to zero were considered unstable under penalization. Internal validation of the penalized model was performed using Harrell's bootstrap optimism-correction procedure (1,000 resamples) to estimate the optimism-corrected AUC and calibration slope. Variance inflation factors (VIFs) were calculated for the predictors retained in the final stepwise multivariable model to assess multicollinearity, with VIF > 5 and VIF > 10 considered indicative of moderate and severe collinearity, respectively. These sensitivity analyses were implemented in Python (version 3.13) using the scikit-learn and statsmodels packages.

The predictive performance of the creatinine-based and cystatin C–based eGFR equations was additionally compared using logistic regression models, the DeLong test, calibration analysis, and reclassification indices (NRI/IDI), with full details provided in the Supplementary Material.

For machine learning analysis, several supervised learning algorithms were developed, including logistic regression (LR), random forest (RF), support vector machine (SVM), extreme gradient boosting (XGB), and light gradient boosting machine (LGBM). The candidate predictors used for model development were pre-specified before machine learning model development based on previous literature and clinical knowledge. Data preprocessing included standardization of numerical variables and one-hot encoding of categorical variables using a column transformer framework. To address class imbalance, the Synthetic Minority Oversampling Technique (SMOTE) was applied. Standardization, one-hot encoding, and SMOTE were combined into a single pipeline object that was fit exclusively on the training folds of each cross-validation split and subsequently applied to the corresponding validation fold, so that no information from the validation fold was used during preprocessing or oversampling.

Model development and hyperparameter optimization were conducted using a nested cross-validation strategy combined with randomized search, in which an outer stratified 5-fold split provided genuine training/test partitions and hyperparameter search was performed within each outer training fold using a further inner stratified 5-fold split. Randomized search (RandomizedSearchCV) evaluated up to 15 candidate hyperparameter combinations per model (the full grid was evaluated for LR, RF, and SVM, whose search spaces contained fewer than 15 combinations), scored by AUC, over the following search spaces: LR, regularization strength C ∈ {0.01, 0.1, 1, 10, 50}; RF, number of trees ∈ {200, 400, 600} and maximum depth ∈ {3, 5, 8, 12}; SVM, C ∈ {0.1, 1, 10} and kernel coefficient gamma ∈ {scale, auto}; and XGB and LGBM, number of trees ∈ {200, 400, 600}, maximum depth ∈ {3, 5, 7}, and learning rate ∈ {0.01, 0.05, 0.1}. Within each outer training fold, the selected model was further recalibrated using Platt scaling (CalibratedClassifierCV) fitted on the same inner 5-fold split. The outer and inner data-splitting procedures and the SMOTE oversampling step were seeded (random_state = 42 and 2024, respectively) to ensure reproducible fold assignments and resampling; the base learners and the random sampling of hyperparameter combinations were run under their default library settings and were not additionally seeded. Machine learning models were implemented in Python (version 3.13) using the scikit-learn, imbalanced-learn, XGB, LGBM, and SHAP libraries. Each outer test fold was held out from both hyperparameter tuning and calibration throughout, so that all reported performance metrics were computed from genuine out-of-fold, out-of-sample predictions. Predictive performance was evaluated using the area under the receiver operating characteristic curve (AUC). Additional performance metrics included sensitivity, specificity, accuracy, *F*1 score, positive predictive value (PPV), negative predictive value (NPV), Brier score, and the area under the precision-recall curve (PR-AUC). The optimal classification threshold was determined by maximizing the Youden index. To assess the robustness of model discrimination, 95% confidence intervals for AUC were estimated using bootstrap resampling. Net reclassification improvement (NRI) and integrated discrimination improvement (IDI) were calculated to compare the predictive performance of machine learning models with logistic regression.

Finally, model interpretability was assessed using Shapley Additive Explanations (SHAP). Feature importance and the direction of each predictor's impact on MACE risk were visualized using SHAP summary plots derived from the XGB model. SHAP values quantify the contribution of each predictor to the model's output and do not, by themselves, imply a causal or biological relationship between a predictor and the outcome. The five machine learning models described above were further evaluated using calibration and decision curve analyses based on the same nested out-of-fold predictions.

## 3 Results

### 3.1 Baseline characteristics stratified by 1-year MACE

**Figure 1:**
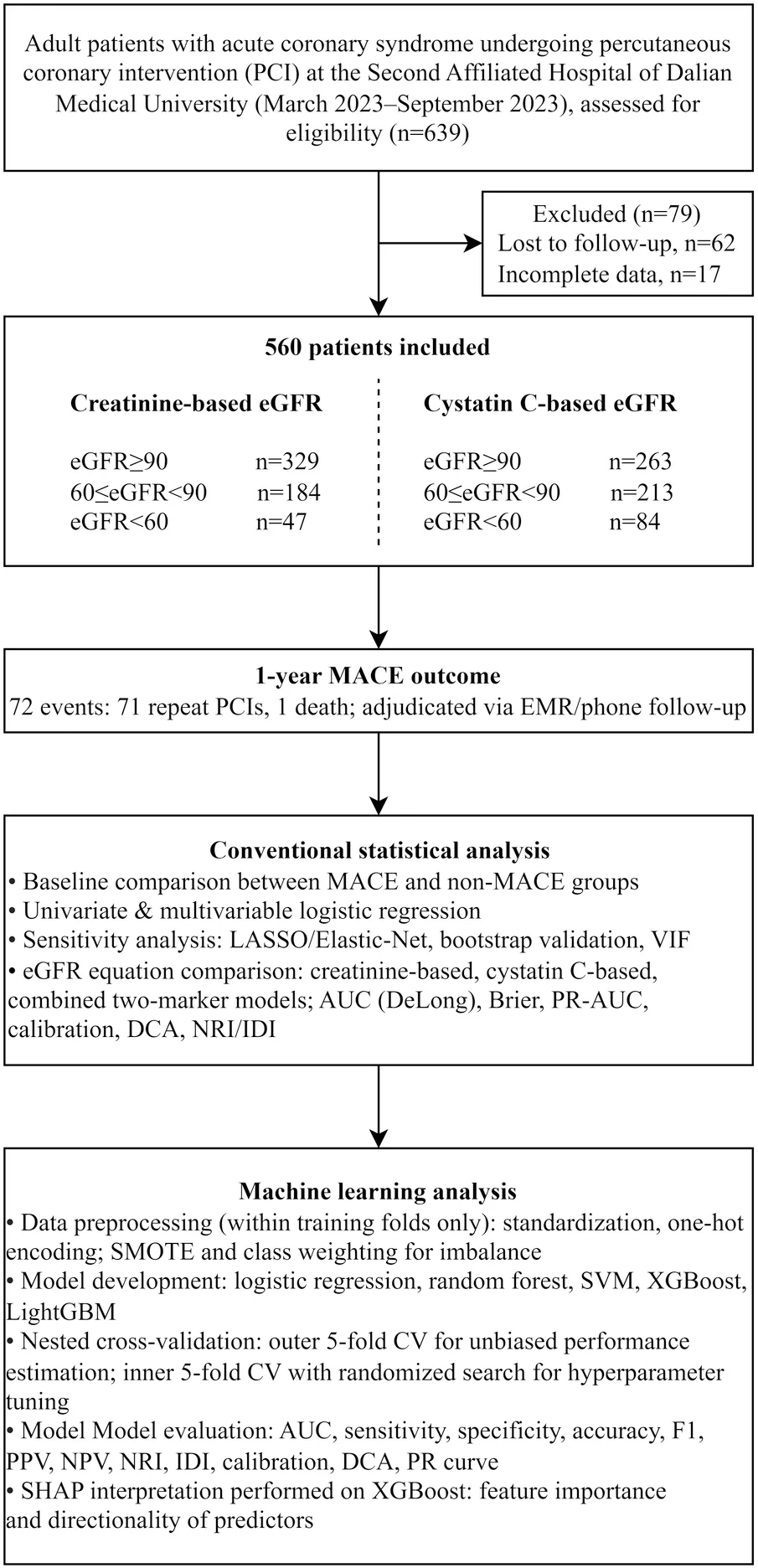
Study flowchart. Flowchart illustrating patient enrollment, renal function classification by creatinine-based and cystatin C–based eGFR, 1-year MACE outcomes, and the parallel conventional statistical and machine learning analysis pipelines used in this study.

A total of 560 patients who underwent PCI were included in the final analysis (Figure 1). During the 1-year follow-up, MACE occurred in 72 patients (12.9%), whereas 488 patients (87.1%) remained free of MACE. Of these 72 events, 71 (98.6%) were repeat PCIs and 1 (1.4%) was a cardiac death; therefore, the composite endpoint in this cohort was driven predominantly by repeat PCI (Table 1). Baseline demographic, clinical, echocardiographic, and laboratory characteristics by MACE status are summarized in Table 2.

**Table 1:** Clinical outcomes during 1-year follow-up (*N* = 560).

Outcome	*n* (%)
No adverse event	381 (68.04)
Repeat percutaneous coronary intervention	71 (12.68)
Angina pectoris	89 (15.89)
Heart failure (hospitalization)	9 (1.61)
Arrhythmia	9 (1.61)
Death	1 (0.18)
1-year MACE (repeat PCI + death)	72 (12.86)

**Table 2:** Baseline characteristics of the study population.

Characteristic	MACE			*p*-value
	Overall	NO	YES	
	*N* = 560	*N* = 488	*N* = 72	
Gender, *n* (%)				0.666^b^
Male	378 (67.50%)	331 (67.83%)	47 (65.28%)	
Female	182 (32.50%)	157 (32.17%)	25 (34.72%)	
Age, median (Q1, Q3)	66 (58, 72)	67 (57, 72)	66 (58, 72)	0.864^a^
Creatinine-based eGFR categories, *n* (%)				0.076^b^
eGFR ≥ 90	329 (58.75%)	289 (59.22%)	40 (55.56%)	
60 ≤ eGFR < 90	184 (32.86%)	163 (33.40%)	21 (29.17%)	
eGFR < 60	47 (8.39%)	36 (7.38%)	11 (15.28%)	
Cystatin C–based eGFR categories, *n* (%)				0.262^b^
eGFR ≥ 90	263 (46.96%)	234 (47.95%)	29 (40.28%)	
60 ≤ eGFR < 90	213 (38.04%)	185 (37.91%)	28 (38.89%)	
eGFR < 60	84 (15.00%)	69 (14.14%)	15 (20.83%)	
Height, median (Q1, Q3)	170 (163, 174)	170 (163, 174)	170 (162, 173)	0.469^a^
Weight, median (Q1, Q3)	70 (64, 80)	70 (65, 80)	70 (62, 80)	0.782^a^
BMI, median (Q1, Q3)	25.3 (23.1, 27.4)	25.3 (23.1, 27.2)	25.4 (22.7, 28.2)	0.563^a^
Acute coronary syndrome, *n* (%)				0.998^b^
Unstable angina	288 (51.43%)	251 (51.43%)	37 (51.39%)	
ST-elevation myocardial infarction	123 (21.96%)	107 (21.93%)	16 (22.22%)	
Non-ST-elevation myocardial infarction	149 (26.61%)	130 (26.64%)	19 (26.39%)	
History of hypertension, *n* (%)	360 (64.29%)	309 (63.32%)	51 (70.83%)	0.214^b^
History of diabetes, *n* (%)	216 (38.57%)	183 (37.50%)	33 (45.83%)	0.175^b^
Smoking history, *n* (%)	232 (41.43%)	205 (42.01%)	27 (37.50%)	0.468^b^
Alcohol consumption history, *n* (%)	75 (13.39%)	64 (13.11%)	11 (15.28%)	0.615^b^
History of stroke, *n* (%)	60 (10.71%)	55 (11.27%)	5 (6.94%)	0.268^b^
Systolic blood pressure, median (Q1, Q3)	135 (121, 151)	135 (121, 150)	139 (123, 152)	0.241^a^
Diastolic blood pressure, median (Q1, Q3)	82 (75, 92)	82 (75, 91)	84 (75, 96)	0.262^a^
Number of diseased vessels, median (Q1, Q3)	3.00 (2.00, 3.00)	3.00 (2.00, 3.00)	3.00 (2.00, 3.00)	0.005^a^
Left ventricular end-diastolic diameter, median (Q1, Q3)	45.8 (43.3, 48.1)	45.4 (43.0, 48.0)	46.8 (44.4, 49.8)	0.009^a^
Interventricular septal thickness, median (Q1, Q3)	10.50 (9.80, 11.50)	10.50 (9.85, 11.50)	10.45 (9.55, 11.50)	0.911^a^
Left ventricular ejection fraction, median (Q1, Q3)	60 (53, 60)	60 (54, 60)	57 (50, 60)	0.010^a^
Blood urea nitrogen, median (Q1, Q3)	6.07 (4.96, 7.17)	6.02 (4.95, 7.08)	6.19 (5.02, 8.60)	0.245^a^
Uric acid, median (Q1, Q3)	350 (290, 412)	350 (289, 407)	353 (294, 426)	0.240^a^
Pro-BNP, median (Q1, Q3)	110 (107, 666)	110 (107, 659)	111 (107, 828)	0.247^a^
Troponin I, median (Q1, Q3)	0 (0, 3)	0 (0, 3)	0 (0, 2)	0.476^a^
CK-MB, median (Q1, Q3)	2 (1, 4)	2 (1, 4)	2 (1, 4)	0.245^a^
Glycated hemoglobin, median (Q1, Q3)	6.30 (5.70, 7.60)	6.20 (5.70, 7.44)	7.00 (6.00, 8.10)	0.006^a^
Total cholesterol, median (Q1, Q3)	4.16 (3.46, 5.05)	4.17 (3.47, 5.05)	4.07 (3.46, 5.01)	0.881^a^
Triglycerides, median (Q1, Q3)	1.42 (1.06, 2.04)	1.43 (1.04, 2.07)	1.36 (1.12, 1.86)	0.823^a^
High-density lipoprotein, median (Q1, Q3)	0.99 (0.85, 1.17)	0.98 (0.84, 1.17)	1.01 (0.89, 1.17)	0.280^a^
Low-density lipoprotein, median (Q1, Q3)	2.34 (1.82, 3.15)	2.35 (1.82, 3.16)	2.28 (1.83, 3.05)	0.851^a^
CRP, median (Q1, Q3)	2 (1, 7)	2 (1, 7)	2 (1, 6)	0.949^a^
White blood cell, median (Q1, Q3)	6.53 (5.37, 8.04)	6.51 (5.37, 8.13)	6.64 (5.21, 7.84)	0.793^a^
Red blood cell, median (Q1, Q3)	4.49 (4.16, 4.82)	4.50 (4.18, 4.82)	4.47 (3.98, 4.83)	0.252^a^
Hemoglobin, median (Q1, Q3)	138 (126, 148)	138 (126, 148)	134 (116, 146)	0.050^a^
Platelet, median (Q1, Q3)	216 (182, 258)	217 (182, 255)	212 (179, 261)	0.831^a^
Alanine aminotransferase, median (Q1, Q3)	22 (15, 32)	21 (15, 32)	24 (17, 34)	0.166^a^
Aspartate aminotransferase, median (Q1, Q3)	21 (17, 33)	21 (17, 34)	21 (16, 28)	0.876^a^

a Wilcoxon rank sum test.

b Pearson's Chi-squared test.

Overall, most demographic and clinical characteristics were comparable between the two groups. Age, sex distribution, body mass index, blood pressure, and lipid profiles did not differ significantly between patients with and without MACE (all *p* > 0.05). Similarly, inflammatory markers, cardiac biomarkers, liver enzymes, and most hematological parameters were largely comparable between groups. In addition, the distribution of acute coronary syndrome subtypes as well as the prevalence of hypertension, diabetes, smoking history, alcohol consumption, and prior stroke did not differ significantly between the two groups.

However, several cardiovascular structural and metabolic parameters were significantly associated with MACE. Patients who experienced MACE had a greater number of diseased coronary vessels than those without MACE (*p* = 0.005). Echocardiographic parameters also differed between groups: the MACE group exhibited a larger left ventricular end-diastolic diameter [46.8 (44.4, 49.8) mm vs. 45.4 (43.0, 48.0) mm, *p* = 0.009] and a lower left ventricular ejection fraction [57 (50, 60)% vs. 60 (54, 60)%, *p* = 0.010]. In addition, glycated hemoglobin levels were significantly higher in the MACE group [7.00 (6.00, 8.10)% vs. 6.20 (5.70, 7.44)%, *p* = 0.006]. Hemoglobin levels were marginally lower in the MACE group, showing borderline statistical significance (*p* = 0.050).

Given the primary objective of this study, particular attention was paid to renal function estimated using different eGFR equations. When renal function was categorized using the creatinine-based eGFR equation, 58.75% of patients were classified as having normal renal function (eGFR ≥ 90 mL/min/1.73 m^2^), 32.86% were classified as having mildly decreased renal function (60–89 mL/min/1.73 m^2^), and 8.39% were classified as having moderate-to-severe renal impairment (eGFR < 60 mL/min/1.73 m^2^). Importantly, the distribution of renal function differed between patients with and without MACE. The proportion of patients with eGFR < 60 mL/min/1.73 m^2^ was notably higher in the MACE group than in the non-MACE group (15.28% vs. 7.38%), representing more than a twofold difference. Conversely, preserved renal function (eGFR ≥ 90 mL/min/1.73 m^2^) was slightly less frequent among patients who experienced MACE (55.56% vs. 59.22%). Although the overall difference did not reach statistical significance (*p* = 0.076), these findings indicate a trend toward poorer renal function among patients who subsequently developed adverse cardiovascular outcomes. When renal function was evaluated using the cystatin C–based eGFR equation, a different classification pattern was observed. In the overall cohort, fewer patients were categorized as having normal renal function (46.96%), while a larger proportion were classified as having moderate-to-severe renal impairment (15.00%). Among patients who experienced MACE, the prevalence of reduced renal function (eGFR < 60 mL/min/1.73 m^2^) was also higher than that observed in patients without MACE (20.83% vs. 14.14%). However, the overall distribution of cystatin C–based eGFR categories did not differ significantly between the two groups (*p* = 0.262).

Notably, the two eGFR equations yielded distinct renal function classification patterns within the study population. Compared with the creatinine-based equation, the cystatin C–based equation classified a substantially larger proportion of patients as having impaired renal function, identifying nearly twice as many patients in the eGFR < 60 mL/min/1.73 m^2^ category (15.00% vs. 8.39%). Despite this broader identification of renal impairment, the relative increase in reduced renal function among patients who developed MACE appeared more pronounced when renal function was categorized using the creatinine-based equation. Overall, patients who experienced MACE were characterized by more extensive coronary artery disease, evidence of adverse cardiac remodeling, poorer glycemic control, and a higher prevalence of reduced renal function.

### 3.2 Logistic regression analysis

**Figure 2:**
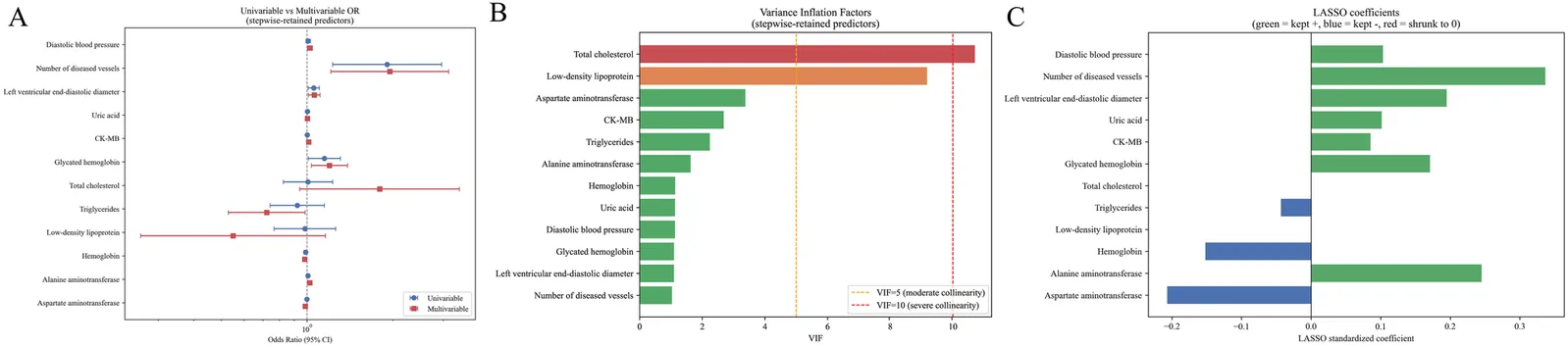
Sensitivity analysis of the stepwise-retained multivariable model. **(A)** Forest plot of univariable and multivariable odds ratios (95% CI) for the 12 predictors retained by backward stepwise selection. **(B)** Variance inflation factors (VIF) for the same predictors; dashed lines denote moderate (VIF = 5) and severe (VIF = 10) collinearity thresholds. **(C)** Standardized coefficients from LASSO logistic regression; a coefficient of 0 indicates the predictor was shrunk out of the model. VIF, variance inflation factor; LASSO, least absolute shrinkage and selection operator.

**Figure 3:**
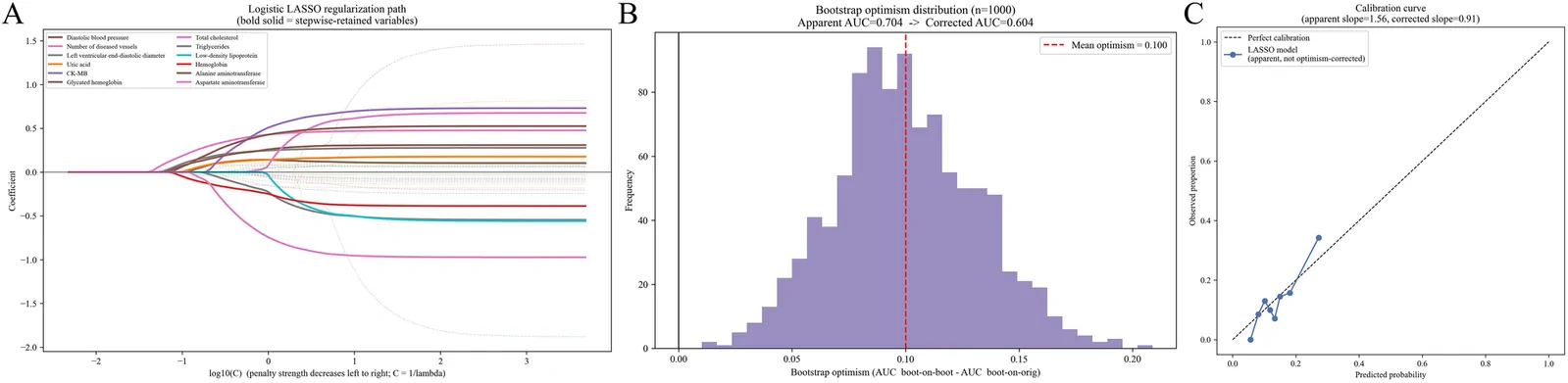
Penalized regression and internal validation of the multivariable model. **(A)** Logistic LASSO regularization path for all 35 candidate predictors across a grid of penalty strengths (*C* = 1/*λ*); bold solid lines denote the 12 stepwise-retained predictors. **(B)** Bootstrap distribution of AUC optimism (1,000 resamples); dashed line indicates the mean optimism used for correction. **(C)** Calibration curve of the LASSO model (apparent, not optimism-corrected). AUC, area under the receiver operating characteristic curve.

To identify potential predictors of 1-year MACE, univariable logistic regression analyses were first performed for all candidate variables. All candidate variables were subsequently entered into a multivariable logistic regression model. The final model was obtained using a backward stepwise selection procedure based on the AIC. Detailed results are presented in Table 3. Because this model retained 12 predictors for 72 events (events-per-variable = 6.0), findings were cross-checked against a parallel penalized regression analysis (Tables 4, 5, Figures 2, 3).

**Table 3:** Logistic regression analysis of predictors of 1-year MACE after PCI.

Characteristic	Univariable					Multivariable				
	*N*	Event N	OR	95% CI	*p*-value	*N*	Event N	OR	95% CI	*p*-value
Gender										
Male	378	47	–	–						
Female	182	25	1.12	0.67, 1.89	0.666					
Age	560	72	1	0.98, 1.02	0.994					
Creatinine-based eGFR categories										
eGFR ≥ 90	329	40	–	–						
60 ≤ eGFR < 90	184	21	0.93	0.53, 1.63	0.803					
eGFR < 60	47	11	2.21	1.04, 4.68	0.039*					
Cystatin C–based eGFR categories										
eGFR ≥ 90	263	29	–	–						
60 ≤ eGFR < 90	213	28	1.22	0.70, 2.13	0.479					
eGFR < 60	84	15	1.75	0.89, 3.46	0.105					
Height	560	72	0.98	0.95, 1.01	0.283					
Weight	560	72	1	0.98, 1.02	0.759					
BMI	560	72	1.02	0.95, 1.09	0.678					
History of hypertension	360	51	1.41	0.82, 2.42	0.216					
History of diabetes	216	33	1.41	0.86, 2.32	0.176					
Smoking history	232	27	0.83	0.50, 1.38	0.469					
Alcohol consumption history	75	11	1.19	0.60, 2.39	0.615					
History of stroke	60	5	0.59	0.23, 1.52	0.273					
Systolic blood pressure	560	72	1.01	1.00, 1.02	0.217					
Diastolic blood pressure	560	72	1.01	0.99, 1.03	0.342	560	72	1.02	1.00, 1.04	0.029*
Number of diseased vessels	560	72	1.92	1.24, 2.99	0.004**	560	72	1.96	1.22, 3.15	0.006**
Left ventricular end-diastolic diameter	560	72	1.06	1.01, 1.10	0.018*	560	72	1.06	1.01, 1.11	0.017*
Interventricular septal thickness	560	72	1.02	0.87, 1.20	0.793					
Left ventricular ejection fraction	560	72	0.97	0.94, 0.99	0.016*					
Blood urea nitrogen	560	72	1.12	1.03, 1.23	0.011*					
Uric acid	560	72	1	1.00, 1.00	0.089	560	72	1	1.00, 1.00	0.096
Pro-BNP	560	72	1	1.00, 1.00	0.672					
Troponin I	560	72	0.99	0.98, 1.01	0.572					
CK-MB	560	72	1	1.00, 1.01	0.817	560	72	1.01	1.00, 1.02	0.007**
Glycated hemoglobin	560	72	1.15	1.01, 1.31	0.032*	560	72	1.2	1.04, 1.39	0.015*
Total cholesterol	560	72	1.01	0.82, 1.23	0.945	560	72	1.8	0.95, 3.43	0.073
Triglycerides	560	72	0.93	0.74, 1.15	0.487	560	72	0.72	0.53, 0.98	0.040*
High-density lipoprotein	560	72	1.61	0.68, 3.81	0.284					
Low-density lipoprotein	560	72	0.98	0.76, 1.26	0.881	560	72	0.55	0.26, 1.16	0.114
CRP	560	72	1	0.99, 1.01	0.799					
White blood cell	560	72	0.97	0.89, 1.06	0.52					
Red blood cell	560	72	0.74	0.48, 1.14	0.178					
Hemoglobin	560	72	0.99	0.97, 1.00	0.055	560	72	0.98	0.97, 0.99	0.007**
Platelet	560	72	1	1.00, 1.00	0.711					
Alanine aminotransferase	560	72	1.01	1.00, 1.01	0.123	560	72	1.02	1.01, 1.03	<0.001***
Aspartate aminotransferase	560	72	1	0.99, 1.00	0.399	560	72	0.99	0.97, 1.00	0.009**

CI, confidence interval; OR, odds ratio. Null deviance = 430; null df = 559; Log-likelihood = −191; AIC = 408; BIC = 464; deviance = 382; residual df = 547; No. Obs. = 560.

* *p* < 0.05.

** *p* < 0.01.

*** *p* < 0.001.

**Table 4:** Sensitivity analysis of the stepwise-retained predictors: univariable and multivariable odds ratios, LASSO standardized coefficients, and variance inflation factors.

Variable	Univariable OR (*p*)	Multivariable OR (*p*)	LASSO standardized coefficient	VIF
Diastolic blood pressure	1.01 (0.328)	1.02 (0.028)	0.103	1.13
Number of diseased vessels	1.91 (0.004)	1.95 (0.006)	0.337	1.03
Left ventricular end-diastolic diameter	1.06 (0.019)	1.06 (0.017)	0.195	1.09
Uric acid	1.00 (0.097)	1.00 (0.100)	0.102	1.13
CK-MB	1.00 (0.826)	1.01 (0.007)	0.085	2.68
Glycated hemoglobin	1.15 (0.034)	1.20 (0.015)	0.171	1.09
Total cholesterol	1.01 (0.941)	1.80 (0.075)	0.000	10.70
Triglycerides	0.92 (0.479)	0.72 (0.040)	−0.043	2.24
Low-density lipoprotein	0.98 (0.893)	0.55 (0.116)	0.000	9.18
Hemoglobin	0.99 (0.053)	0.98 (0.007)	−0.152	1.13
Alanine aminotransferase	1.01 (0.128)	1.02 (0.001)	0.245	1.62
Aspartate aminotransferase	1.00 (0.392)	0.99 (0.009)	−0.207	3.37

Univariable and multivariable estimates were independently recomputed using Python (statsmodels) for this sensitivity analysis; minor numerical differences relative to Table 3 (computed in R) reflect software-specific implementation and do not indicate divergent conclusions. LASSO (10-fold cross-validated) retained 18 of 35 candidate predictors. Among the four variables flagged as unexpected in the unpenalized model: CK-MB retained only a small LASSO coefficient and was shrunk to zero under elastic net (Table 5), indicating instability; alanine aminotransferase and aspartate aminotransferase retained larger, opposite-signed coefficients, consistent with statistical suppression given their moderate correlation (*r* = 0.60) rather than independent opposing effects; triglycerides were shrunk close to zero under LASSO and further attenuated under elastic net, suggesting that its unpenalized significance (adjusted OR 0.72, *p* = 0.040) reflects a selection artifact rather than a robust association. Total cholesterol and LDL cholesterol, both with wide confidence intervals in the unpenalized model, were shrunk entirely to zero under LASSO.

**Table 5:** Standardized coefficients of the candidate predictors retained by LASSO and elastic-net logistic regression applied to the same 35 candidate predictors used in the univariable analysis.

Variable	LASSO standardized coefficient	Elastic-net standardized coefficient
Number of diseased vessels	0.337	0.270
Alanine aminotransferase	0.245	0.167
Aspartate aminotransferase	−0.207	−0.070
Left ventricular end-diastolic diameter	0.195	0.149
Glycated hemoglobin	0.171	0.132
Hemoglobin	−0.152	−0.114
History of stroke (yes vs. no)	−0.111	−0.078
Diastolic blood pressure	0.103	0.053
Uric acid	0.102	0.069
CK-MB	0.085	–
High-density lipoprotein	0.080	0.062
Blood urea nitrogen	0.076	0.077
Height	−0.067	−0.037
Systolic blood pressure	0.045	0.044
Creatinine-based eGFR category (<60 vs. ≥90 mL/min/1.73 m^2^)	0.044	0.045
Triglycerides	−0.043	−0.005
Left ventricular ejection fraction	−0.035	−0.047
Age	−0.004	–

LASSO and elastic-net (*α* = 0.5) models were fitted to the same 35 candidate predictors, with the regularization parameter in each selected by 10-fold cross-validation. LASSO retained 18 predictors; elastic net retained 16, a subset of the LASSO-selected set. The remaining 17 predictors were shrunk to zero under both methods and omitted here. CK-MB and age, both retained by LASSO, were shrunk to zero under elastic net. Elastic-net coefficients were generally smaller than LASSO coefficients, as expected under the added L2 penalty, but concordant in direction and ranking. As standardized coefficients from penalized regression lack *p*-values or confidence intervals under standard maximum-likelihood theory, these estimates should be interpreted as exploratory rather than confirmatory.

In the univariable logistic regression analysis, several clinical and laboratory variables were significantly associated with the occurrence of MACE, while a few others reached significance only after multivariable adjustment. Among hemodynamic parameters, diastolic blood pressure emerged as an independent predictor of adverse outcomes after multivariable adjustment (*p* = 0.029). Indicators reflecting the severity of coronary artery disease were also strongly associated with MACE; specifically, the number of diseased coronary vessels was significantly positively associated with the risk of events. Echocardiographic parameters reflecting cardiac structure were also important predictors. Left ventricular end-diastolic diameter was significantly associated with MACE, suggesting that ventricular remodeling may contribute to adverse cardiovascular outcomes after PCI. Several biochemical markers were additionally associated with MACE after multivariable adjustment. CK-MB, a marker of myocardial injury, was retained in the stepwise model (multivariable *p* = 0.007), although its penalized-regression coefficient was small (Table 5). Metabolic indicators such as glycated hemoglobin were also associated with MACE after multivariable adjustment, indicating a possible role for metabolic disturbances in long-term cardiovascular prognosis; triglyceride levels were also retained in the stepwise model, but their coefficient was shrunk close to zero under LASSO, so this association should be considered unstable. Hematological parameters were also relevant; hemoglobin levels were associated with the risk of MACE after multivariable adjustment, suggesting anemia may contribute to poorer outcomes. In addition, liver function indicators such as alanine aminotransferase and aspartate aminotransferase were retained in the stepwise model, though their opposite-signed coefficients may reflect statistical suppression rather than independent effects (Table 5). Several other biochemical variables, such as uric acid, total cholesterol, and low-density lipoprotein cholesterol, showed a trend toward association with adverse outcomes, although these did not reach statistical significance. Left ventricular ejection fraction (*p* = 0.016) and blood urea nitrogen (*p* = 0.011) were also statistically significant in univariable analysis but were not retained after AIC–based backward stepwise selection.

Variables identified in the univariable analysis were subsequently included in the multivariable logistic regression model, and the final model was determined using an AIC–based backward stepwise selection procedure. In the multivariable model, several variables remained independently associated with MACE. The number of diseased coronary vessels remained an independent predictor, indicating that patients with more extensive coronary artery disease faced a significantly higher risk of adverse cardiovascular events following PCI. Left ventricular end-diastolic diameter also remained independently associated with MACE, highlighting the role of structural cardiac remodeling in determining long-term prognosis. Diastolic blood pressure also remained significantly associated with MACE after adjustment (adjusted OR 1.02, *p* = 0.029), despite not reaching significance in the univariable analysis (*p* = 0.342)—a pattern suggestive of statistical suppression by other correlated predictors retained in the model. Biochemical markers reflecting myocardial injury and metabolic status were also retained in the final model. CK-MB and triglyceride coefficients were both shrunk close to zero under LASSO (Table 4), indicating these two associations were less stable than the others. Glycated hemoglobin was retained in the stepwise model, consistent with a possible role for glycemic control. Among hematological parameters, hemoglobin levels were associated with the risk of MACE in the adjusted model. Liver enzyme levels, including alanine aminotransferase and aspartate aminotransferase, were retained in the stepwise model, though their opposite-signed coefficients may reflect statistical suppression rather than independent biological effects.

Given that the primary objective of this study was to compare the predictive performance of different eGFR equations, renal function estimated using both creatinine-based and cystatin C–based formulas was further examined. When renal function was categorized using the creatinine-based eGFR equation, patients with moderate-to-severe renal impairment (eGFR < 60 mL/min/1.73 m^2^) demonstrated a significantly higher risk of MACE compared with those with preserved renal function (eGFR ≥ 90 mL/min/1.73 m^2^) in univariable analysis (*p* = 0.039); however, this variable was not retained in the final model after AIC–based backward stepwise selection, and therefore did not remain an independent predictor in the multivariable analysis. In contrast, individuals with mildly decreased renal function (60 ≤ eGFR < 90 mL/min/1.73 m^2^) did not show a statistically significant increase in risk. These findings suggest that the risk of adverse cardiovascular events becomes more pronounced once renal dysfunction progresses to a moderate or severe stage. When renal function was evaluated using the cystatin C–based eGFR equation, a similar pattern was observed. Patients with lower eGFR categories exhibited a higher risk of MACE compared with those with preserved renal function. In particular, individuals with cystatin C–based eGFR < 60 mL/min/1.73 m^2^ demonstrated a higher estimated risk of adverse events, whereas those with mildly reduced renal function showed an intermediate risk profile. However, none of the cystatin C–based eGFR categories were retained in the final model after AIC–based backward stepwise selection, and therefore they did not remain independent predictors in the multivariable analysis. Overall, the logistic regression analyses suggest that renal dysfunction is associated with an increased risk of 1-year MACE after PCI, and this association is particularly evident when renal impairment is defined using the creatinine-based eGFR equation. The findings also indicate that moderate-to-severe reductions in eGFR may have greater prognostic relevance than mild decreases in renal function.

Given that the final multivariable model retained 12 predictors while only 72 MACE events were observed, the events-per-variable (EPV) ratio was 6.0, below the conventional threshold of 10 generally recommended to minimize overfitting (EPV based on the same 35 candidate predictors used in the univariable analysis, was 2.06). To assess the stability of the multivariable findings under this constraint, penalized (LASSO) logistic regression, bootstrap internal validation, and variance inflation factor (VIF) analysis were performed as sensitivity analyses. Internal validation using bootstrap resampling (1,000 iterations) indicated an apparent AUC of 0.704 that decreased to an optimism-corrected AUC of 0.604 (mean optimism = 0.100), consistent with a degree of overfitting in the original stepwise model. Total cholesterol and low-density lipoprotein cholesterol showed evidence of moderate-to-severe multicollinearity (VIF = 10.70 and 9.18, respectively) and their coefficients were shrunk to zero under LASSO. CK-MB and triglycerides retained only small non-zero LASSO coefficients and were unstable across penalization methods (Tables 4, 5); alanine aminotransferase and aspartate aminotransferase retained larger, opposite-signed coefficients that may reflect statistical suppression. These parallel penalized-regression results indicate that the CK-MB and triglyceride associations in particular were less stable than the other stepwise-retained predictors. Full results, including univariable, multivariable, and LASSO coefficients for all stepwise-retained predictors, are provided in Tables 4, 5 and Figures 2, 3.

Direct comparison of the two eGFR equations showed similarly modest discrimination and calibration among the creatinine-based, cystatin C–based, and combined two-marker models (AUC 0.546–0.561), with no statistically significant difference by the DeLong test (all *p* > 0.4) and minimal incremental value (IDI ≤ 0.005; Supplementary Table S1); decision curve analysis is shown in Supplementary Figure S1.

### 3.3 Machine learning model

**Figure 4:**
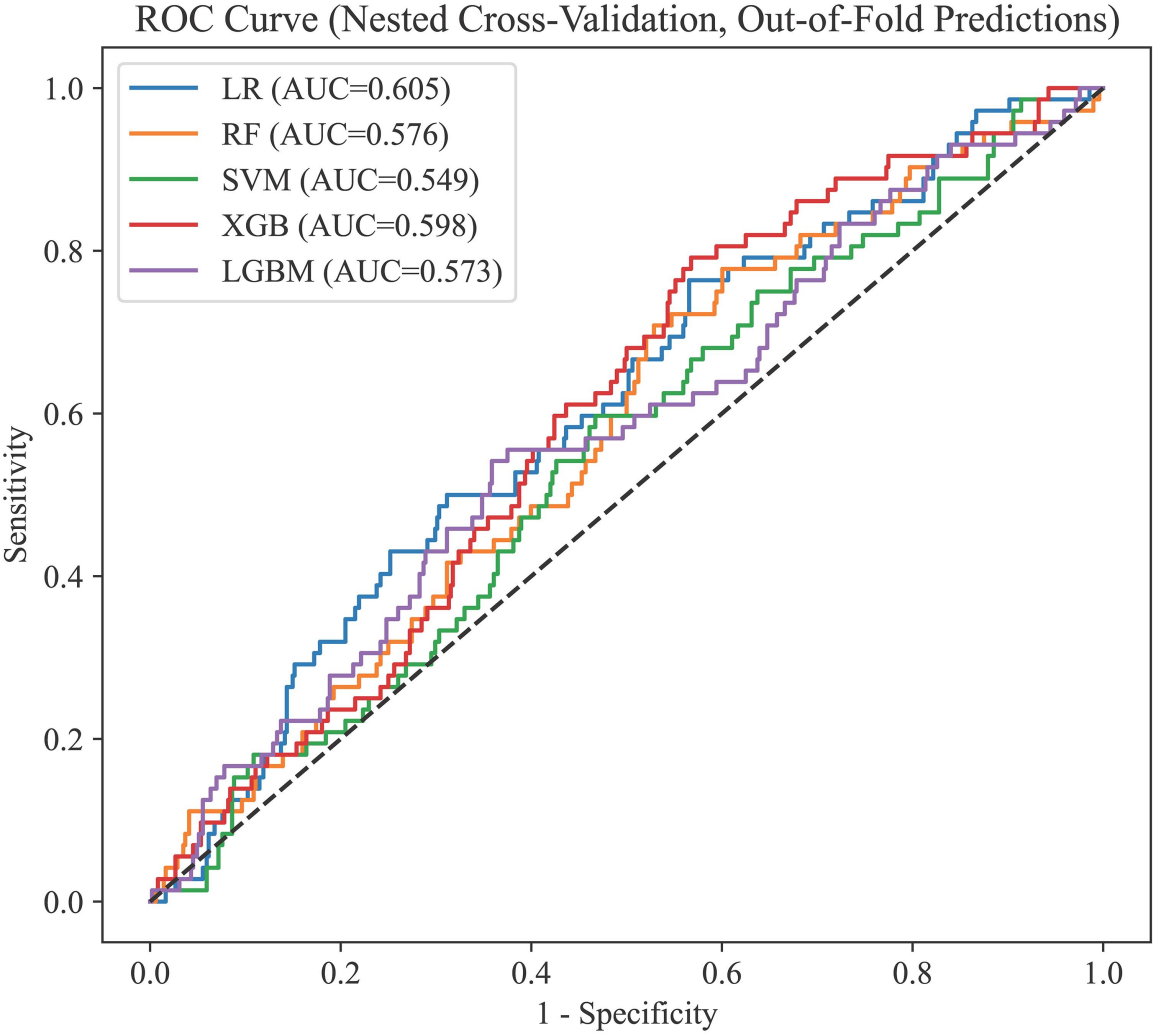
Receiver operating characteristic (ROC) curves of different models for predicting 1-year major adverse cardiovascular events (MACE) after percutaneous coronary intervention (PCI). ROC curves comparing the predictive performance of five models for 1-year MACE after PCI. The evaluated models included logistic regression (LR), random forest (RF), support vector machine (SVM), extreme gradient boosting (XGB), and light gradient boosting machine (LGBM). The AUC values were 0.605 for LR, 0.576 for RF, 0.549 for SVM, 0.598 for XGB, and 0.573 for LGBM.

To further evaluate the predictive performance of clinical and laboratory variables for 1-year MACE, several machine learning algorithms were constructed. The candidate predictors used for model development were pre-specified based on previous literature and clinical knowledge. Five machine learning algorithms were implemented, including LR, RF, SVM, XGB, and LGBM. The predictive performance of the models was assessed using receiver operating characteristic (ROC) curve analysis and multiple classification metrics, including sensitivity, specificity, accuracy, *F*1 score, negative predictive value (NPV), and positive predictive value (PPV). The ROC curves of the five models are presented in Figure 4, and the detailed performance metrics are summarized in Table 6.

**Table 6:** Performance of machine learning models for predicting 1-year MACE after PCI.

Model	AUC (95% CI)	Sensitivity	Specificity	Accuracy	*F*1	NPV	PPV	NRI	IDI
LR	0.605 (0.532–0.673)	76.389	43.443	47.679	27.295	92.576	16.616	–	–
RF	0.576 (0.509–0.642)	70.833	47.131	50.179	26.772	91.634	16.505	−0.111	−0.004
SVM	0.549 (0.478–0.619)	59.722	53.279	54.107	25.073	89.965	15.867	−0.241	−0.007
XGB	0.598 (0.529–0.663)	79.167	43.238	47.857	28.079	93.363	17.066	−0.033	−0.002
LGBM	0.573 (0.502–0.645)	54.167	64.139	62.857	27.273	90.462	18.224	−0.161	−0.005

Among the evaluated algorithms, based on nested cross-validation out-of-fold predictions, the logistic regression model demonstrated the highest discriminative ability, with an AUC of 0.605 (95% CI: 0.532–0.673), followed by XGB (AUC 0.598, 95% CI: 0.529–0.663). The RF and LGBM models showed comparable, lower performance (AUC 0.576 and 0.573, respectively), and the SVM model showed the lowest performance (AUC 0.549, 95% CI: 0.478–0.619). Overall, the predictive performance of the models was modest, with AUC values ranging from 0.549 to 0.605. Regarding classification performance, the XGB model achieved the highest sensitivity (79.17%), although its specificity was relatively limited (43.24%); the LGBM model demonstrated the highest specificity (64.14%) at the cost of lower sensitivity (54.17%). Accuracy ranged from 47.68% to 62.86%, and *F*1 scores were modest across all models, reflecting the imbalanced outcome distribution. Reclassification analysis showed negative NRI/IDI values for all machine learning models relative to logistic regression (NRI −0.033 to −0.241; IDI −0.002 to −0.007), indicating that none of the more complex algorithms improved risk stratification beyond logistic regression once evaluated on genuinely out-of-sample predictions.

Extended evaluation of the five machine learning models, including calibration, precision-recall, and decision curve analyses, is presented in Supplementary Table S2 and Supplementary Figure S2. Brier scores (0.111–0.112) remained close to the no-skill baseline expected at the observed 12.9% event rate, indicating limited absolute predictive gain despite reasonable calibration slopes in some models (Supplementary Table S2).

**Figure 5:**
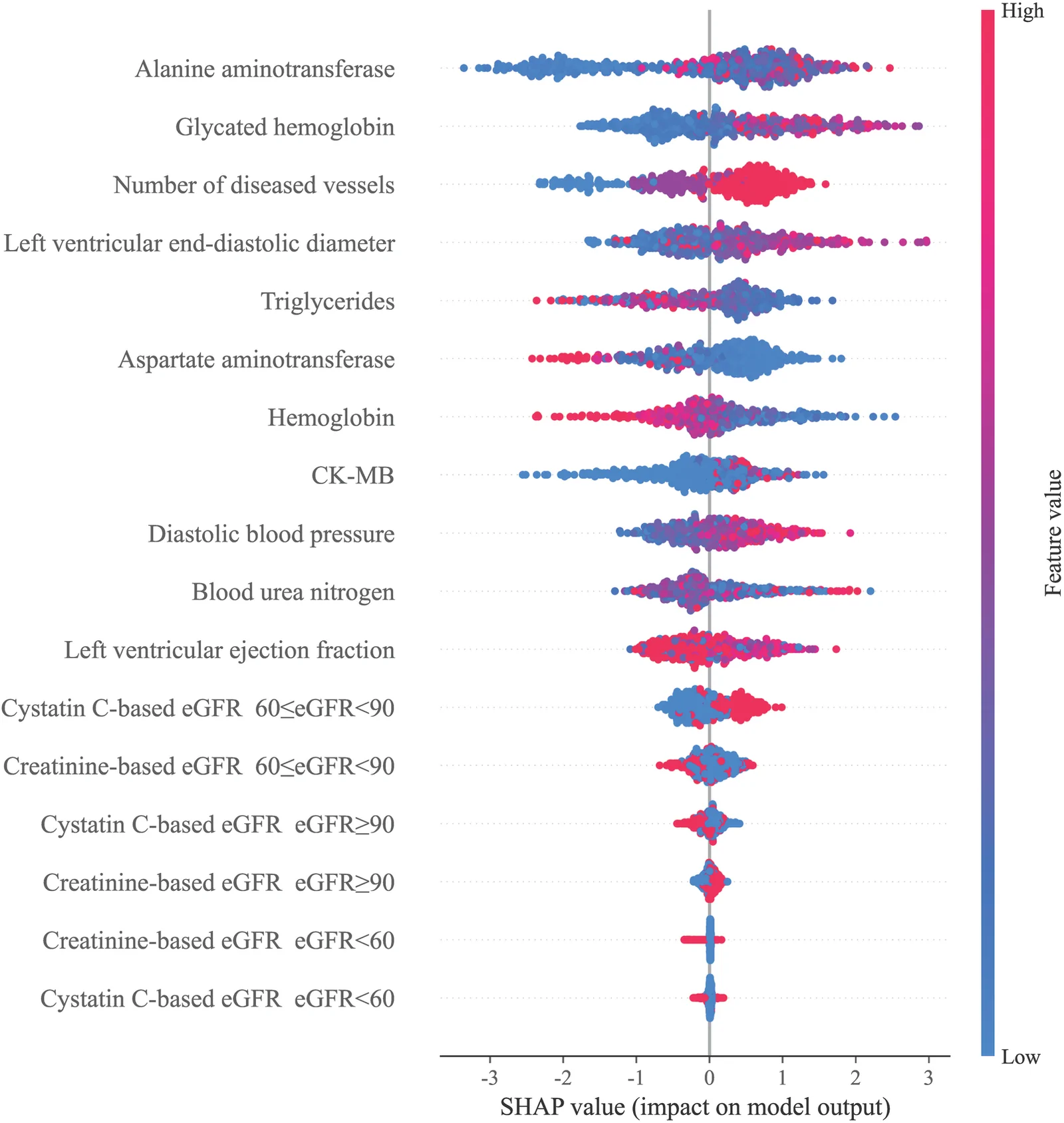
SHAP summary plot showing the contribution of clinical variables to the prediction of 1-year major adverse cardiovascular events (MACE) after percutaneous coronary intervention (PCI). SHAP (Shapley Additive Explanations) summary plot illustrating the relative importance of predictors in the XGB model for 1-year MACE. Each dot represents an individual patient. The *x*-axis indicates the SHAP value, reflecting the impact of each variable on model output, while color represents the feature value (red: high, blue: low). eGFR, estimated glomerular filtration rate; CK-MB, creatine kinase-MB.

To enhance the interpretability of the machine learning model, SHAP analysis was performed using the XGB model. Although logistic regression achieved the highest AUC, XGB was selected for SHAP interpretation because, among the remaining tree-based and kernel-based algorithms, it achieved the highest AUC (0.598) with a relatively balanced sensitivity–specificity profile, and because its tree-based structure is well suited to capturing nonlinear and interaction effects among predictors—information not readily available from the logistic regression coefficients, which already provide direct interpretability through odds ratios. The SHAP summary plot is presented in Figure 5. In this plot, each point represents an individual observation, and the horizontal position reflects the SHAP value, which indicates the impact of the feature on the model's predictions. The color gradient represents the magnitude of the feature value, with red indicating higher feature values and blue indicating lower values. The SHAP analysis showed that several cardiovascular and metabolic variables exerted the greatest influence on model predictions. Variables such as alanine aminotransferase, glycated hemoglobin, the number of diseased vessels, left ventricular end-diastolic diameter, triglycerides, aspartate aminotransferase, hemoglobin, CK-MB, diastolic blood pressure, blood urea nitrogen, and left ventricular ejection fraction ranked among the most important predictors in the model. In general, higher values of diastolic blood pressure, blood urea nitrogen, CK-MB, and glycated hemoglobin (red points) tended to be associated with positive SHAP values, indicating an increased predicted risk of MACE. Conversely, higher triglyceride and left ventricular ejection fraction values were predominantly associated with negative SHAP values, indicating a lower model-predicted risk of MACE.

Renal function indicators derived from both creatinine-based and cystatin C–based eGFR equations were also included in the model. Because each eGFR category was entered into the model as a one-hot encoded binary variable, SHAP values for these one-hot–encoded eGFR categories should not be interpreted as a linear regression–style contrast against a single dropped reference category. Rather, consistent with the Shapley value framework, the SHAP value assigned to each binary eGFR category reflects that feature's own contribution to the difference between the model's prediction for an individual patient and the model's expected (average) output across the cohort, rather than a continuous dose–response relationship with eGFR. Although these features ranked lower in the overall SHAP importance ranking compared with several cardiovascular and metabolic variables, their SHAP value distributions still demonstrated meaningful contributions to risk prediction. In particular, observations corresponding to reduced eGFR categories tended to shift toward positive SHAP values, suggesting that impaired renal function contributes to an increased predicted probability of MACE. These findings indicate that while renal dysfunction is associated with adverse cardiovascular outcomes, other clinical and biochemical variables may have a greater impact on machine learning–based risk prediction in this cohort.

## 4 Discussion

### 4.1 Principal findings of the present study

In this retrospective cohort study of patients undergoing PCI, we evaluated the prognostic value of renal function estimated using different eGFR equations for predicting 1-year MACE. Several key findings emerged from the present analysis. First, during the 1-year follow-up, MACE occurred in 12.9% of the study population, indicating that a substantial proportion of patients remain at risk of adverse cardiovascular outcomes even after successful coronary revascularization. Because 71 of the 72 MACE events (98.6%) were repeat PCIs and only 1 was a cardiac death, the study findings should be interpreted primarily as predictors of repeat PCI rather than a balanced composite cardiovascular endpoint. This observation is consistent with the concept of “residual cardiovascular risk,” which remains an important clinical challenge in contemporary interventional cardiology (20, 21).

Second, renal dysfunction demonstrated heterogeneous associations with clinical outcomes depending on the equation used to estimate eGFR. Moderate-to-severe renal impairment defined using the creatinine-based eGFR equation was significantly associated with an increased risk of MACE in univariable analysis, although it was not retained in the final multivariable model after AIC–based backward stepwise selection, whereas renal function categorized by the cystatin C–based equation showed a weaker and statistically non-significant relationship with clinical outcomes. These findings suggest that although both equations are designed to estimate the same physiological parameter—glomerular filtration rate—they may capture different aspects of renal function and therefore differ in their ability to stratify cardiovascular risk in patients undergoing PCI.

### 4.2 Clinical determinants of MACE after PCI

In addition to renal function, several cardiovascular and metabolic factors were identified as important determinants of adverse outcomes after PCI in this study. These findings are consistent with the well-established understanding that post-PCI prognosis is influenced by a complex interplay of coronary disease burden, myocardial functional status, and systemic metabolic conditions (22–25).

One of the most prominent predictors observed in the present cohort was the number of diseased coronary vessels, which was significantly higher among patients who experienced MACE. Multivessel coronary artery disease is widely recognized as a marker of more extensive atherosclerotic burden and has been consistently associated with worse clinical outcomes following coronary revascularization (26, 27). Patients with diffuse coronary involvement often exhibit more complex plaque morphology, greater plaque instability, and a higher likelihood of residual ischemia despite PCI (28). This broader vascular involvement may predispose patients to recurrent ischemic events, repeat revascularization, and cardiovascular mortality during follow-up.

Cardiac structural and functional parameters were also significant determinants of clinical outcomes. Patients who developed MACE exhibited a larger left ventricular end-diastolic diameter and a lower left ventricular ejection fraction compared with those without events. These findings suggest that ventricular remodeling and impaired myocardial contractility may substantially influence long-term prognosis after PCI. Left ventricular dilation often represents a chronic adaptive response to myocardial injury or sustained hemodynamic stress. Over time, this remodeling process may lead to increased wall stress, progressive myocardial dysfunction, and the development of heart failure. Similarly, reduced ejection fraction reflects diminished systolic performance and has long been recognized as one of the strongest predictors of cardiovascular morbidity and mortality in patients with coronary artery disease (29).

Metabolic abnormalities also contributed to the risk of MACE in this population. Higher glycated hemoglobin levels were observed among patients who experienced adverse outcomes, highlighting the potential impact of impaired glycemic control on cardiovascular prognosis after PCI. Chronic hyperglycemia promotes a wide range of pathophysiological processes that accelerate atherosclerosis, including endothelial dysfunction, oxidative stress, and plaque instability, while also frequently coexisting with dyslipidemia, insulin resistance, and diffuse microvascular disease that further compromise revascularization outcomes. Taken together, these findings emphasize that the development of MACE after PCI is driven by multiple interrelated factors. The burden of coronary atherosclerosis, the structural and functional integrity of the myocardium, and systemic metabolic disturbances all contribute to the overall cardiovascular risk profile. Within this complex clinical context, renal dysfunction represents a critical additional dimension of risk that may interact with other pathophysiological processes to influence long-term outcomes.

### 4.3 Comparative predictive value of different eGFR equations for MACE after PCI

A central objective of this study was to compare the prognostic performance of different equations used to estimate glomerular filtration rate in predicting adverse cardiovascular outcomes after PCI. Renal dysfunction is widely recognized as a major risk factor for cardiovascular morbidity and mortality, and reduced eGFR has consistently been associated with an increased risk of cardiovascular events in patients with coronary artery disease (4, 30, 31). However, different biomarkers used for estimating eGFR may capture distinct physiological and pathological aspects of kidney function, potentially leading to variation in cardiovascular risk stratification.

In the baseline analysis of this cohort, the creatinine-based and cystatin C–based equations demonstrated markedly different patterns of renal function classification. The cystatin C–based equation identified a substantially larger proportion of patients with reduced renal function, particularly in the category of moderate-to-severe impairment. This observation is consistent with previous studies indicating that cystatin C may be more sensitive than creatinine in detecting early declines in glomerular filtration (31, 32). Unlike serum creatinine, which is strongly influenced by muscle mass, dietary intake, and demographic characteristics, cystatin C is produced by all nucleated cells at a relatively constant rate and is less affected by body composition. Consequently, cystatin C–based equations often detect subtle reductions in renal function that may not yet be captured by changes in serum creatinine levels.

Despite identifying a larger number of patients with reduced kidney function, the cystatin C–based eGFR equation did not demonstrate a stronger association with clinical outcomes in the present study. In contrast, moderate-to-severe renal impairment defined by the creatinine-based equation was significantly associated with an increased risk of 1-year MACE in univariable analysis, although this association was not retained after multivariable adjustment. This apparent discrepancy suggests that the two equations may capture different dimensions of renal physiology that do not necessarily translate into equivalent prognostic information for cardiovascular outcomes. One possible explanation is that creatinine-based eGFR may better reflect chronic structural kidney damage in patients with established cardiovascular disease, whereas cystatin C may be more sensitive to short-term physiological fluctuations or non-renal influences such as systemic inflammation. Because inflammatory activity is frequently elevated in patients with coronary artery disease, cystatin C concentrations may partly reflect inflammatory status rather than pure glomerular filtration capacity, potentially weakening its specificity as a prognostic marker in this setting.

Another factor that may have contributed to the observed differences is the overall distribution of renal function within the study population. In the present cohort, the majority of patients had relatively preserved kidney function, with only a small proportion exhibiting an eGFR below 60 mL/min/1.73 m^2^. Under such circumstances, the clinical impact of minor differences in eGFR estimation may be limited. Indeed, our results suggest that the prognostic significance of renal dysfunction becomes more evident only when kidney function declines to the level of moderate or severe impairment. Patients with mildly reduced renal function did not exhibit a significantly increased risk of MACE compared with those with normal renal function, whereas individuals with eGFR below 60 mL/min/1.73 m^2^ showed a significantly elevated risk. This finding is consistent with the concept that clinically meaningful cardiovascular consequences of kidney disease often emerge once renal impairment reaches a threshold at which systemic pathophysiological alterations become pronounced.

The relationship between advanced renal dysfunction and cardiovascular risk is supported by several well-established biological mechanisms (33, 34), including neurohormonal activation, endothelial dysfunction, and accelerated atherosclerosis, which may help explain why patients with more advanced renal dysfunction are particularly susceptible to adverse cardiovascular outcomes after coronary revascularization.

The contribution of renal function to cardiovascular risk prediction was further explored using machine learning models and SHAP-based model interpretation. Notably, although renal function indicators were included in the predictive models, their relative importance was less than that of several cardiovascular and metabolic variables such as left ventricular ejection fraction, blood urea nitrogen, diastolic blood pressure, and markers of myocardial injury. This finding suggests that among patients undergoing PCI, renal dysfunction may act as an important but secondary contributor to risk prediction when compared with factors directly reflecting cardiovascular, metabolic, and coronary disease severity. Nevertheless, the SHAP value distributions indicated that lower eGFR levels tended to shift model predictions toward a higher probability of adverse outcomes, supporting the notion that impaired renal function exerts a meaningful—albeit moderate—effect on cardiovascular risk stratification.

### 4.4 Interpretation of machine learning results

In addition to traditional statistical analyses, this study further explored the predictive performance of several machine learning algorithms for identifying patients at risk of 1-year MACE after PCI. Overall, the predictive performance of the evaluated models, assessed using nested cross-validation to avoid tuning-related optimism, was modest, with area under the ROC curve ranging from approximately 0.55–0.60. Interestingly, the conventional logistic regression model demonstrated the highest discriminative ability among the tested algorithms, whereas more complex machine learning models such as RF, SVM, and gradient boosting methods did not provide a substantial improvement in predictive accuracy.

Several factors may explain why machine learning approaches did not outperform the traditional logistic regression model in the present study. First, the sample size of the cohort was modest, with 560 patients and a limited number of outcome events. Machine learning algorithms generally benefit from large-scale datasets characterized by complex nonlinear interactions between variables (35). In smaller datasets, these algorithms may be more prone to overfitting and may not fully exploit their theoretical advantages. Second, the number of predictors included in the model was relatively limited and primarily consisted of conventional clinical and laboratory variables. When the relationships between predictors and outcomes are predominantly linear or monotonic, simpler statistical models such as logistic regression may perform comparably to or even better than more complex machine learning approaches. Third, many of the important predictors identified in this cohort—such as cardiac functional parameters and biochemical markers—have well-established linear associations with cardiovascular outcomes, further reducing the potential incremental benefit of nonlinear modeling techniques.

To enhance the interpretability of the machine learning model, SHAP analysis was applied to quantify the relative contribution of each predictor to model output (36, 37). The SHAP results revealed that several cardiovascular and metabolic variables exerted the strongest influence on predicted risk. Specifically, variables including alanine aminotransferase, glycated hemoglobin, the number of diseased vessels, left ventricular end-diastolic diameter, and triglycerides ranked among the most important predictors in the model. Notably, many of these variables were also identified as relevant predictors in the logistic regression analysis, highlighting a robust concordance between machine learning–based interpretation and traditional statistical findings. This concordance enhances the credibility of the model and suggests that the identified predictors are consistent, non-random contributors to model output rather than algorithm-specific artifacts; SHAP values reflect each predictor's contribution to the model's prediction and do not by themselves establish a biological or causal relationship with the outcome.

Renal function indicators derived from both creatinine-based and cystatin C–based eGFR equations were also included in the machine learning models. Although these variables ranked lower in overall feature importance compared with several cardiovascular and metabolic parameters, their SHAP value distributions still demonstrated a meaningful contribution to model predictions. Specifically, lower eGFR values were generally associated with positive SHAP values, indicating an increased predicted probability of MACE. These findings suggest that renal dysfunction contributes to cardiovascular risk prediction but may not be the dominant determinant of outcomes in patients undergoing PCI. Instead, renal impairment appears to interact with other systemic and cardiovascular factors to influence the overall risk profile.

### 4.5 Clinical implications

The findings of this study have several clinical implications. Renal function assessment, particularly using creatinine-based eGFR, remains a relevant component of cardiovascular risk stratification in patients undergoing PCI, even though its added value beyond conventional cardiovascular and metabolic variables was modest in this cohort. The differing performance of creatinine-based and cystatin C–based equations suggests that the two biomarkers may provide complementary rather than interchangeable information, and that the choice of eGFR equation may influence which patients are identified as high-risk. Finally, the comparable performance of logistic regression and machine learning models in this moderately sized dataset indicates that conventional statistical approaches remain a practical option for cardiovascular risk prediction in similar clinical settings, without necessarily requiring more complex modeling techniques.

### 4.6 Limitations

This study has several limitations that should be considered when interpreting these findings. First, it was a single-center retrospective analysis, which may introduce selection bias and limit generalizability. Because of this single-center design, the observed difference in prognostic association between creatinine-based and cystatin C–based eGFR should be regarded as a hypothesis-generating finding rather than evidence of definitive clinical superiority of either equation. Second, although the overall sample size was moderate, the number of MACE events was relatively small, which may affect statistical power and model stability; to address this, internal validation of the multivariable model was performed using bootstrap resampling, which indicated a degree of overfitting (optimism-corrected AUC of 0.604 vs. an apparent AUC of 0.704; Figure 3), underscoring the need for external validation before clinical application. Third, renal function assessment was restricted to a single baseline measurement, precluding the evaluation of longitudinal changes or subclinical fluctuations during follow-up or around the time of PCI. In addition, because of the limited number of MACE events, eGFR was categorized into only three groups rather than the full KDIGO classification, and the eGFR < 60 mL/min/1.73 m^2^ category encompassed a broad and heterogeneous range of renal impairment severity, which may have limited the granularity of the observed associations. Fourth, several periprocedural variables that may influence the risk of MACE, including the specific coronary vessel(s) treated and the completeness of revascularization (i.e., complete revascularization vs. culprit-vessel-only PCI), were not systematically recorded and could not be accounted for in the present analysis. Despite these limitations, this study benefited from a rigorously curated cohort with complete-case data across all analyzed variables and extensive internal validation, including bootstrap resampling, calibration assessment, and comparison across multiple machine learning algorithms, which strengthen the robustness of the comparative findings. Larger prospective multicenter studies are needed to validate these findings and to clarify their generalizability to broader populations.

## 5 Conclusion

In conclusion, this study evaluated the comparative predictive performance of creatinine-based and cystatin C–based eGFR equations for 1-year MACE in patients undergoing PCI. Renal dysfunction was common in this population and associated with an increased risk of adverse cardiovascular outcomes. The cystatin C–based equation identified a substantially larger proportion of patients with impaired renal function, whereas moderate-to-severe renal dysfunction defined by the creatinine-based equation was significantly associated with an increased risk of 1-year MACE in univariable analysis. Creatinine-based eGFR was not retained as an independent predictor after AIC–based multivariable adjustment, but it contributed to MACE risk prediction in the machine learning models. As this was a single-center retrospective study, these findings should be regarded as hypothesis-generating rather than establishing definitive clinical superiority of either eGFR equation, and external validation in independent, prospective, multicenter cohorts is warranted before clinical application.

Machine learning models exhibited modest predictive performance overall and failed to surpass the traditional logistic regression model. Feature importance analysis further indicated that cardiovascular functional parameters and metabolic markers were the primary contributors to risk prediction, while renal function indicators provided complementary prognostic value.

These findings highlight the importance of integrating renal function assessment into cardiovascular risk stratification after PCI. In particular, although creatinine-based eGFR alone showed limited discriminative ability (AUC ≈ 0.55) and was not retained as an independent predictor in the adjusted multivariable model, it may still provide complementary information when considered alongside other clinical and laboratory parameters within a broader risk-assessment framework. Further large-scale prospective studies are warranted to validate these results and explore whether combining multiple renal biomarkers could further improve risk prediction.

## Data Availability

The datasets presented in this article are not readily available because the data that support the findings of this study are available on request from the corresponding author. The data are not publicly available due to privacy or ethical restrictions. Requests to access the datasets should be directed to Zhaohong Geng, gengzhaohong@dmu.edu.cn.

## References

[1] Neumann F-J Sousa-Uva M Ahlsson A Alfonso F Banning AP Benedetto U. 2018 ESC/EACTS guidelines on myocardial revascularization. Eur Heart J. (2019) 40:87–165. 10.1093/eurheartj/ehy394 30165437

[2] Serruys PW Morice M-C Kappetein AP Colombo A Holmes DR Mack MJ. Percutaneous coronary intervention versus coronary-artery bypass grafting for severe coronary artery disease. N Engl J Med. (2009) 360:961–72. 10.1056/NEJMoa0804626 19228612

[3] Stone GW Kappetein AP Sabik JF Pocock SJ Morice M-C Puskas J. Five-year outcomes after PCI or CABG for left main coronary disease. N Engl J Med. (2019) 381:1820–30. 10.1056/NEJMoa1909406 31562798

[4] Go AS Chertow GM Fan D McCulloch CE Hsu CY. Chronic kidney disease and the risks of death, cardiovascular events, and hospitalization. N Engl J Med. (2004) 351:1296–305. 10.1056/NEJMoa041031 15385656

[5] Ndumele CE Neeland IJ Tuttle KR Chow SL Mathew RO Khan SS. A synopsis of the evidence for the science and clinical management of cardiovascular-kidney-metabolic (CKM) syndrome: a scientific statement from the American heart association. Circulation. (2023) 148:1636–64. 10.1161/cir.0000000000001186 37807920

[6] Di Lullo L House A Gorini A Santoboni A Russo D Ronco C. Chronic kidney disease and cardiovascular complications. Heart Fail Rev. (2015) 20:259–72. 10.1007/s10741-014-9460-9 25344016

[7] Düsing P Zietzer A Goody PR Hosen MR Kurts C Nickenig G. Vascular pathologies in chronic kidney disease: pathophysiological mechanisms and novel therapeutic approaches. J Mol Med. (2021) 99:335–48. 10.1007/s00109-021-02037-7 PMC7900031 33481059

[8] Guedeney P Sorrentino S Vogel B Baber U Claessen BE Mehran R. Assessing and minimizing the risk of percutaneous coronary intervention in patients with chronic kidney disease. Expert Rev Cardiovasc Ther. (2018) 16:825–35. 10.1080/14779072.2018.1526082 30324814

[9] Shin D Galougahi KK Singh M Caron E Cannata M Ciftcikal Y. Coronary artery disease and percutaneous coronary intervention in patients with severe chronic kidney disease. Prog Cardiovasc Dis. (2025) 88:75–9. 10.1016/j.pcad.2024.12.004 39761789

[10] Stevens PE Ahmed SB Carrero JJ Foster B Francis A Hall RK. KDIGO 2024 clinical practice guideline for the evaluation and management of chronic kidney disease. Kidney Int. (2024) 105:S117–314. 10.1016/j.kint.2023.10.018 38490803

[11] Levey AS Stevens LA Schmid CH Zhang YL Castro AF Feldman HI. A new equation to estimate glomerular filtration rate. Ann Intern Med. (2009) 150:604–12. 10.7326/0003-4819-150-9-200905050-00006 19414839 PMC2763564

[12] Shlipak MG Matsushita K Ärnlöv J Inker LA Katz R Polkinghorne KR. Cystatin C versus creatinine in determining risk based on kidney function. N Engl J Med. (2013) 369:932–43. 10.1056/NEJMoa1214234 24004120 PMC3993094

[13] Stevens LA Coresh J Schmid CH Feldman HI Froissart M Kusek J. Estimating GFR using serum cystatin C alone and in combination with serum creatinine: a pooled analysis of 3,418 individuals with CKD. Am J Kid Dis. (2008) 51:395–406. 10.1053/j.ajkd.2007.11.018 18295055 PMC2390827

[14] Delanaye P Cavalier E Cristol JP Delanghe JR. Calibration and precision of serum creatinine and plasma cystatin C measurement: impact on the estimation of glomerular filtration rate. J Nephrol. (2014) 27:467–75. 10.1007/s40620-014-0087-7 24711159

[15] Pottel H Delanaye P Schaeffner E Dubourg L Eriksen BO Melsom T. Estimating glomerular filtration rate for the full age spectrum from serum creatinine and cystatin C. Nephrol Dial Transplant. (2017) 32:497–507. 10.1093/ndt/gfw425 28089986 PMC5837496

[16] Wang Z Gu Y Huang L Liu S Chen Q Yang Y. Construction of machine learning diagnostic models for cardiovascular pan-disease based on blood routine and biochemical detection data. Cardiovasc Diabetol. (2024) 23:351. 10.1186/s12933-024-02439-0 39342281 PMC11439295

[17] Zhu H Qiao S Zhao D Wang K Wang B Niu Y. Machine learning model for cardiovascular disease prediction in patients with chronic kidney disease. Front Endocrinol. (2024) 15:1390729. 10.3389/fendo.2024.1390729 PMC11165240 38863928

[18] Ma Y-C Zuo L Chen J-H Luo Q Yu X-Q Li Y. Modified glomerular filtration rate estimating equation for Chinese patients with chronic kidney disease. J Am Soc Nephrol. (2006) 17:2937–44. 10.1681/asn.2006040368 16988059

[19] Inker LA Schmid CH Tighiouart H Eckfeldt JH Feldman HI Greene T. Estimating glomerular filtration rate from serum creatinine and cystatin C. N Engl J Med. (2012) 367:20–9. 10.1056/NEJMoa1114248 22762315 PMC4398023

[20] Gomez-Delgado F Raya-Cruz M Katsiki N Delgado-Lista J Perez-Martinez P. Residual cardiovascular risk: when should we treat it? Eur J Intern Med. (2024) 120:17–24. 10.1016/j.ejim.2023.10.013 37845117

[21] Tucker B Vaidya K Cochran BJ Patel S. Inflammation during percutaneous coronary intervention-prognostic value, mechanisms and therapeutic targets. Cells. (2021) 10:1391. 10.3390/cells10061391 34199975 PMC8230292

[22] Gragnano F Van Klaveren D Heg D Räber L Krucoff MW Raposeiras-Roubín S. Derivation and validation of the PRECISE-HBR score to predict bleeding after percutaneous coronary intervention. Circulation. (2025) 151:343–55. 10.1161/circulationaha.124.072009 39462482

[23] Alkhouli M Alqahtani F Tarabishy A Sandhu G Rihal CS. Incidence, predictors, and outcomes of acute ischemic stroke following percutaneous coronary intervention. JACC Cardiovasc Interv. (2019) 12:1497–506. 10.1016/j.jcin.2019.04.015 31395220

[24] Kosmidou I Shahim B Dressler O Redfors B Morice M-C Puskas JD. Incidence, predictors, and impact of hospital readmission after revascularization for left main coronary disease. J Am Coll Cardiol. (2024) 83:1073–81. 10.1016/j.jacc.2024.01.012 38479955

[25] Deng W Wang D Wan Y Lai S Ding Y Wang X. Prediction models for major adverse cardiovascular events after percutaneous coronary intervention: a systematic review. Front Cardiovasc Med. (2024) 10:1287434. 10.3389/fcvm.2023.1287434 38259313 PMC10800829

[26] Head SJ Milojevic M Daemen J Ahn J-M Boersma E Christiansen EH. Mortality after coronary artery bypass grafting versus percutaneous coronary intervention with stenting for coronary artery disease: a pooled analysis of individual patient data. Lancet. (2018) 391:939–48. 10.1016/s0140-6736(18)30423-9 29478841

[27] Park S Kim W Rha S-W Choi BG Hahn J-Y Park S-H. Effectiveness and safety of biodegradable polymer-coated everolimus-eluting stent in multivessel coronary artery disease in routine clinical practice: a Korean multicenter, prospective, and observational study. J Korean Med Sci. (2026) 41:e85. 10.3346/jkms.2026.41.e85 41807025 PMC12976635

[28] Bentzon JF Otsuka F Virmani R Falk E. Mechanisms of plaque formation and rupture. Circ Res. (2014) 114:1852–66. 10.1161/circresaha.114.302721 24902970

[29] Liu Y Song J Wang W Zhang K Qi Y Yang J. Association of ejection fraction with mortality and cardiovascular events in patients with coronary artery disease. ESC Heart Fail. (2022) 9:3461–8. 10.1002/ehf2.14063 35866195 PMC9715855

[30] Sarnak MJ Levey AS Schoolwerth AC Coresh J Culleton B Hamm LL. Kidney disease as a risk factor for development of cardiovascular disease: a statement from the American heart association councils on kidney in cardiovascular disease, high blood pressure research, clinical cardiology, and epidemiology and prevention. Hypertension. (2003) 42:1050–65. 10.1161/01.HYP.0000102971.85504.7c 14604997

[31] Matsushita K Ballew SH Wang AY Kalyesubula R Schaeffner E Agarwal R. Epidemiology and risk of cardiovascular disease in populations with chronic kidney disease. Nat Rev Nephrol. (2022) 18:696–707. 10.1038/s41581-022-00616-6 36104509

[32] Dharnidharka VR Kwon C Stevens G. Serum cystatin C is superior to serum creatinine as a marker of kidney function: a meta-analysis. Am J Kidney Dis. (2002) 40:221–6. 10.1053/ajkd.2002.34487 12148093

[33] Marx-Schütt K Cherney DZI Jankowski J Matsushita K Nardone M Marx N. Cardiovascular disease in chronic kidney disease. Eur Heart J. (2025) 46:2148–60. 10.1093/eurheartj/ehaf167 PMC12167664 40196891

[34] Baaten C Vondenhoff S Noels H. Endothelial cell dysfunction and increased cardiovascular risk in patients with chronic kidney disease. Circ Res. (2023) 132:970–92. 10.1161/circresaha.123.321752 37053275 PMC10097498

[35] Beam AL Kohane IS. Big data and machine learning in health care. J Am Med Assoc. (2018) 319:1317–8. 10.1001/jama.2017.18391 29532063

[36] Qi X Wang S Fang C Jia J Lin L Yuan T. Machine learning and SHAP value interpretation for predicting comorbidity of cardiovascular disease and cancer with dietary antioxidants. Redox Biol. (2025) 79:103470. 10.1016/j.redox.2024.103470 39700695 PMC11729017

[37] Li X Zhao Y Zhang D Kuang L Huang H Chen W. Development of an interpretable machine learning model associated with heavy metals’ exposure to identify coronary heart disease among US adults via SHAP: findings of the US NHANES from 2003 to 2018. Chemosphere. (2023) 311:137039. 10.1016/j.chemosphere.2022.137039 36342026

