# Supplementary material for: Comparative predictive performance of different eGFR equations for 1-year major adverse cardiovascular events after percutaneous coronary intervention in patients with acute coronary syndrome
Source: Front Cardiovasc Med. 2026 Sep 9;13:1863081. doi: 10.3389/fcvm.2026.1863081 (PMC13597273; doi:10.3389/fcvm.2026.1863081)
Supplement: Supplementary file 1 [file Supplementaryfile1.docx]

Supplementary Material

# 1 Supplementary Figures and Tables

## 1.1 Supplementary Figures

**Supplementary Figure S1.** Decision curve analysis (DCA) of the creatinine-based eGFR, cystatin C–based eGFR, and combined two-marker models for predicting 1-year MACE after PCI.

The x-axis represents threshold probability (1%–50%), and the y-axis represents net benefit. Colored lines show the net benefit of the creatinine-based eGFR, cystatin C–based eGFR, and combined two-marker models; the gray dashed line represents the "treat all" strategy, and the solid black horizontal line at zero represents the "treat none" strategy. All three models provided net benefit over both reference strategies only within a narrow range of low threshold probabilities (approximately <15%), with negligible separation among the three curves across the plotted range.

**Supplementary Figure S2.** Decision curve analysis (DCA) of the five machine learning models for predicting 1-year MACE after PCI.

The x-axis represents threshold probability (1%–50%), and the y-axis represents net benefit. Colored lines show the net benefit of the logistic regression (LR), random forest (RF), support vector machine (SVM), extreme gradient boosting (XGB), and light gradient boosting machine (LGBM) models, based on nested cross-validation out-of-fold, recalibrated predicted probabilities; the gray dashed line represents the "treat-all" strategy, and the solid black horizontal line at zero represents the "treat-none" strategy. All five models tracked closely with the treat-all strategy at threshold probabilities below approximately 10%. As the threshold approached and exceeded the outcome prevalence (12.9%), net benefit for the models declined more slowly than the treat-all strategy, such that from a threshold of roughly 11%–13% onward, all models exceeded the treat-all strategy. Relative to the treat-none strategy, net benefit across the ~13%–25% range was inconsistent: models were intermittently and modestly above zero, interspersed with brief dips below zero (e.g., SVM fell to approximately -0.009 near a threshold of 20%). Beyond this range, net benefit for RF, SVM, XGB, and LGBM converged toward the treat-none line, while LR showed a small but persistent negative net benefit (approximately -0.001 to -0.007) at thresholds above 30%, indicating no consistent net clinical advantage over a treat-none strategy in that range. Overall, the clinical-utility gain over default strategies was confined to a narrow band of threshold probabilities, consistent with the models' modest discrimination.

## 1.2 Supplementary Tables

**Supplementary Table S1.** Discrimination, calibration, and precision-recall performance of the creatinine-based eGFR, cystatin C–based eGFR, and combined two-marker models for predicting 1-year MACE after PCI.

| **Model** | **AUC (95% CI)** | **Brier score** | **PR-AUC** | **Calibration intercept** | **Calibration slope** |
| --- | --- | --- | --- | --- | --- |
| Creatinine–based eGFR | 0.546 (0.484–0.613) | 0.111 | 0.149 | 0.282 | 1.149 |
| Cystatin C–based eGFR | 0.550 (0.486–0.615) | 0.112 | 0.145 | 0.122 | 1.064 |
| Combined two-marker model | 0.561 (0.492–0.633) | 0.111 | 0.161 | 0.229 | 1.121 |

Three logistic regression models — incorporating creatinine-based eGFR alone, cystatin C–based eGFR alone, and a combined two-marker model incorporating both variables — were fitted using ordinary (unweighted, non-resampled) logistic regression on the entire cohort to obtain properly calibrated predicted probabilities. Baseline ("no-skill") reference values were Brier score = 0.112 and PR-AUC = 0.129 (outcome prevalence); calibration intercept = 0 and slope = 1 indicate perfect calibration. Pairwise DeLong tests showed no statistically significant difference in AUC among the three models (all p > 0.4: creatinine- vs. cystatin C–based, p = 0.925; creatinine-based vs. combined two-marker model, p = 0.710; cystatin C–based vs. combined two-marker model, p = 0.429). Including both eGFR variables in a single model yielded a modest net reclassification improvement over either single-marker model (NRI 0.109–0.162) but negligible integrated discrimination improvement (IDI ≤0.005), indicating minimal true gain in discriminatory ability. Abbreviations: AUC = area under the receiver operating characteristic curve; CI = confidence interval; PR-AUC = area under the precision-recall curve; NRI = net reclassification improvement; IDI = integrated discrimination improvement.

**Supplementary Table S2.** Calibration and precision-recall performance of the five machine learning models for predicting 1-year MACE after PCI.

| **Model** | **Brier score** | **PR-AUC** | **Calibration intercept** | **Calibration slope** |
| --- | --- | --- | --- | --- |
| LR | 0.112 | 0.167 | -0.528 | 0.724 |
| RF | 0.111 | 0.164 | -0.119 | 0.946 |
| SVM | 0.112 | 0.149 | -0.860 | 0.554 |
| XGB | 0.111 | 0.169 | 0.575 | 1.319 |
| LGBM | 0.111 | 0.167 | -0.112 | 0.949 |

Model performance was evaluated using nested cross-validation: hyperparameter tuning and probability recalibration (Platt scaling) were performed exclusively within each outer training fold, and all values shown are derived from the resulting out-of-fold, out-of-sample predictions. Baseline ("no-skill") reference values were Brier score = 0.112 and PR-AUC = 0.129 (outcome prevalence); calibration intercept = 0 and slope = 1 indicate perfect calibration. Calibration was closest to ideal for RF and LGBM (slopes 0.946 and 0.949; intercepts -0.119 and -0.112) and showed greater residual deviation for LR, SVM, and XGB (slopes 0.724, 0.554, and 1.319; intercepts -0.528, -0.860, and 0.575, respectively). Brier scores (0.111–0.112) were essentially equivalent to the no-skill baseline, and PR-AUC values (0.149–0.169) were only modestly above the prevalence-based baseline, together indicating limited absolute predictive gain. Abbreviations: LR = logistic regression; RF = random forest; SVM = support vector machine; XGB = extreme gradient boosting; LGBM = light gradient boosting machine; AUC = area under the receiver operating characteristic curve; CI = confidence interval; PR-AUC = area under the precision-recall curve.
